# HINT1 Is Involved in the Chronic Mild Stress Elicited Oxidative Stress and Apoptosis Through the PKC ε/ALDH-2/4HNE Pathway in Prefrontal Cortex of Rats

**DOI:** 10.3389/fnbeh.2021.690344

**Published:** 2021-06-09

**Authors:** Fei Liu, Ying-ying Dong, Gang Lei, Yuan Zhou, Peng Liu, Yong-hui Dang

**Affiliations:** ^1^Key Laboratory of Shaanxi Province for Craniofacial Precision Medicine Research, College of Stomatology, Xi’an Jiaotong University, Xi’an, China; ^2^College of Medicine & Forensics, Xi’an Jiaotong University Health Science Center, Xi’an, China; ^3^Department of Psychiatry, First Affiliated Hospital of Xi’an Jiaotong University Health Science Center, Xi’an, China; ^4^Department of Disaster Psychiatry, Graduate School of Medicine, Tohoku University, Sendai, Japan

**Keywords:** HINT1, chronic mild stress, oxidative stress, apoptosis, prefrontal cortex

## Abstract

Major depressive disorder (MDD) is a severe, highly heterogeneous, and life-threatening psychiatric disease which affects up to 21% of the population worldwide. A new hypothesis suggests that the mitochondrial dysfunction causing oxidative stress (OS) and dysregulation of apoptosis in brain might be one of the key pathophysiological factors in MDD. Histidine triad nucleotide binding protein 1 (HINT1), which was first supposed to be protein kinase C (PKC) inhibitor, has been gradually demonstrated to be involved in diverse neuropsychiatric diseases. It still remains elusive that how HINT1 involves in depression. The present study utilized a rat model exposed to chronic mild stress (CMS) to explore the involvement of HINT1 in depression. Face validity, construct validity and predictive validity of CMS model were comprehensive evaluated in this study. Behavioral tests including sucrose preference test, open field test, and elevated plus maze and forced swimming test revealed that stressed rats displayed elevated level of anxiety and depression compared with the controls. CMS rats showed a significant decrease of superoxide dismutase, and a marked increase malondialdehyde levels in prefrontal cortex (PFC). We also found the CMS rats had elevated expression of HINT1, decreased levels of phosphorylated-PKC ε and aldehyde dehydrogenase-two (ALDH-2), and accumulated 4-hydroxynonenal (4HNE) in PFC. Moreover, CMS increased the levels of cleaved caspase-3 and Bax, and decreased the level of Bcl-2 in PFC. The alterations in behavior and molecule were prevented by antidepressant venlafaxine. These results demonstrated that HINT1 was involved in the CMS elicited OS and apoptosis in PFC, probably through the PKC ε/ALDH-2/4HNE pathway. The results suggest that the suppression of HINT1 might have potential as a novel therapeutic strategy for depression.

## Introduction

Major depressive disorder (MDD), according to the Diagnostic and Statistical Manual of Mental Disorders-5 (DSM-5), is a severe, highly heterogeneous and life-threatening psychiatric disease which manifests psychological, behavioral and physiological symptoms (Liu et al., 2017a). Although it affects up to 21% of the population worldwide (Maes et al., 2009), the exact mechanisms of MDD have not been identified yet. The traditional hypotheses of pathogenesis on MDD include hippocampal BDNF insufficient and apoptosis of neurons (Belmaker and Agam, 2008; Krishnan and Nestler, 2008; Kupfer et al., 2012). The prefrontal cortex (PFC), as a significant nerve center of behavior management, is one of the most important areas in brain associated with depression (Liu et al., 2017b; Tepfer et al., 2021).

A new hypothesis suggests that the mitochondrial dysfunction causing oxidative stress (OS) and dysregulation of apoptosis in brain might be one of the key pathophysiological factors in MDD (Lucca et al., 2009; Jimenez-Fernandez et al., 2015; Lindqvist et al., 2017). OS is a form of cellular stress that represents an imbalance between generation of reactive oxygen species (ROS) and the defensive ability of antioxidant in biological system (Jimenez-Fernandez et al., 2015). Normally, a limited number of ROS are produced in cellular metabolic processes and are quickly eliminated to prevent cell damage. However, under pathological conditions, such as immoderate exposure to ultraviolet light, noxious chemicals, and injury, will lead to the excessive accumulation of ROS (Lindqvist et al., 2017). Once the endogenous antioxidant system is not capable of removing excessive ROS, cellular impairment is caused by damaging DNA, proteins, and lipids (Lindqvist et al., 2017). Mitochondrial aldehyde dehydrogenase-two (ALDH-2), which is enriched in brain, plays an important role in maintaining the function of mitochondria (Xia et al., 2020), and prevents against OS and apoptosis (Liu et al., 2017c; Wang et al., 2017). The activation or suppression of ALDH-2 could be bi-directionally regulated via protein kinase C (PKC) ε phosphorylation in the brain, and also associated with metabolizing other biogenic aldehydes such as 4-hydroxynonenal (4HNE) or malondialdehyde (MDA) (Li et al., 2017; Goma et al., 2021).

Histidine triad nucleotide binding protein 1 (HINT1), which belongs to the histidine triad (HIT) enzyme superfamily, was first supposed to be PKC inhibitor (Dang et al., 2017). During the past decades, HINT1 has been gradually demonstrated to be involved in diverse neuropsychiatric diseases (Dang et al., 2017). To be specific, several rodent behavioral researches by our group have revealed that HINT1 may play an essential role in the action of mechanisms of schizophrenia (Lei et al., 2020), peripheral pain sensitivity (Liu et al., 2016), drug addiction (Liu et al., 2020, 2021), as well as emotion-related behavior (Sun et al., 2017; Zhou et al., 2018). So far, only Ge et al. (2015) has reported HINT1 is up-regulated in the model of depression. Nevertheless, it still remains elusive that how HINT1 involves in MDD.

The chronic mild stress (CMS) model has been widely accepting as a rodent model of depression (Willner, 1997; Ma et al., 2011). It refers to exposing rodents to a variety of unpredictable mild stressors during a protracted time course (Dang et al., 2017). Currently, the CMS model was not only developed to mimic various behavioral, neuroendocrine and neuroimmune alterations, which are similar to those observed in depressive disorder patients but it is also suitable for evaluating the effects of antidepressant (Matchkov et al., 2015; Zhang et al., 2015). Recently, we found that CMS during adolescence could potentiate anxiety and depressive-like behaviors in male rats (unpublished).

Venlafaxine, an antidepressant that blocks both serotonin and norepinephrine transporters, has been demonstrated significantly more effective than selective serotonin reuptake inhibitors (SSIRs) in depression treatment (Stahl et al., 2002). Thus, in the present study, we adopted the modified CMS model in rats to explore the following questions: (1) whether HINT1 is involved in the PKCε/ALDH-2/4HNE pathway under chronic stress in PFC; (2) whether there is an interrelation between 4HNE cumulation and apoptosis-related proteins in PFC under chronic stress; (3) the intervening effects of antidepressant venlafaxine was also evaluated.

## Materials and Methods

### Animals

A total of 40 male SD rats (age: 3 weeks; average weight: 60 ± 5 g) were purchased from the experimental animal center of Shaanxi Province (Xi’an, China). All rats were reared under standard animal housing conditions in 12 h light-dark cycle (lights on at 7:00 A.M.) and 22 ± 1°C. The experimental procedures were approved by the Animal Care and Use Committee of Xi’an Jiaotong University. All efforts were made to minimize the number of animals used and their suffering.

Baseline consumption of 1% sucrose solution was measured three times in the first week (rats age 3 weeks). Eight rats that failed to acquire stable sucrose preference were excluded. The remaining 32 rats were randomly divided into two groups. A total of 16 rats in the stressed group were bred individually in cages with CMS procedure, while the other 16 rats were bred on normal conditions (four per cage). Then rats in those two groups were divided into two subsets, with each subset receiving venlafaxine or normal saline (NS).

**FIGURE 1:**
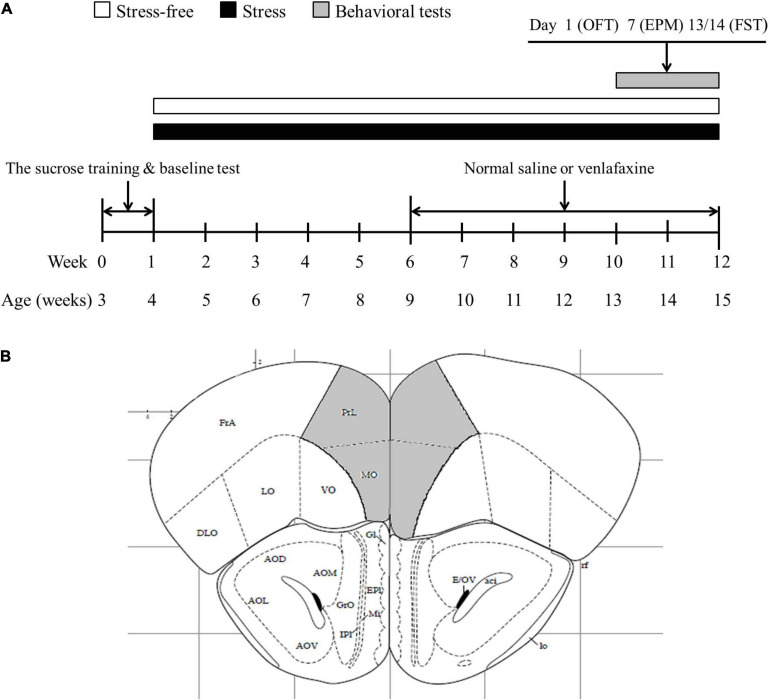
Experimental design and the extents of the investigated encephalic regions. **(A)** Experimental design. The rats were provided 1 week of sucrose training and baseline test. The rats in CMS group were exposed to stress, while rats in control group were bred on normal conditions. Five weeks later, venlafaxine or normal saline were injected intraperitoneally at a fixed time daily. Behavioral tests were performed at the end of stress session. OFT, open field test; EPM, elevated; FST, forced swimming test. **(B)** The extents of the prefrontal cortex (PFC) (the areas in gray). Bregma + 2.80 mm.

The CMS procedure of rats was applied for 11 weeks (rats age 4–15 weeks). The administration of venlafaxine or NS was lasted for 6 weeks (rats age 9–15 weeks). The behavioral tests were carried out during the last 2 weeks (rats age 13–15 weeks). Three kinds of tests were applied on the 1st, 7th, 13th, and 14th day of the behavioral tests weeks, individually. The behavioral test was regarded as a kind of stressor, so on the very day of behavioral test, no other stress would be applied on rats. The experimental design is presented in Figure 1.

### CMS Regimen

The CMS model is established by mimicking the situation of depression in human, which consists of exposing rodents to a period of unpredictable, mild stressors. Details of this procedure, including reliability and validity of the model, have been verified elsewhere (Hill et al., 2012). The CMS regimen was based on our previously described protocol (Ma et al., 2011, 2016). Briefly, rats in the stressed group received a variety of stressors, including 45° cage tilting, empty cage, soiled cage, cage-switching, water deprivation, food deprivation, tail squeezing, immobilization stress, continuous overnight illumination, and inversion of the light/dark cycle. These stressors were applied randomly during both light and dark periods.

### Venlafaxine Treatment

Subset division of stressed and control rats generated ultimately four groups (*n* = 8 for each group): Group 1, control + NS; Group 2, control + venlafaxine; Group 3, CMS + NS; and Group 4, CMS + venlafaxine. Venlafaxine [10 mg/kg body weight (Xing et al., 2013)] and NS were injected intraperitoneally at a fixed time daily for 6 weeks. The administration of NS or venlafaxine was not interrupt during the behavioral tests.

### Behavioral Tests

#### Sucrose Preference Test

When the CMS procedure was initiated, the amount of 1% sucrose consumed between 10:00 and 11:00 A.M. was measured every Saturday. We used the sucrose preference test (SPT) as described by Willner (1997) and Chen et al. (2020). Fourteen hours before SPT, all rats were deprived of water. For the grouped housed control rats, they were separated individually in cages 2 h before the tests. Two bottles, one containing 1% sucrose solution and the other containing tap water, were weighed and delivered to each rat for 1 h. The position of the two bottles was randomly determined. After the test, the grouped housed control rats were bred on normal conditions again (four per cage) according to the marks. Sucrose and water consumptions (g) were measured. Sucrose preference was calculated as follows:

$$sucrosepreference=\frac{sucroseconsumption}{sucrosesolutionconsumption+waterconsumption}\times 100\%$$

#### Open Field Test

We used the open field test (OFT) as described by Chen et al. (2020). The apparatus consisted of a square box with dimensions of 100 cm × 100 cm × 45 cm. Rats were placed individually into the chamber under a dim light (25 lx) for 1 h and the tracks were recorded by a video tracking system (SMART, Panlab SL, Barcelona, Spain). Time spent in the central area, entries in central area, distance traveled in the first 10 min and total distance were recorded. The chamber was thoroughly cleaned with 75% ethanol between tests.

#### Elevated Plus Maze

We used the elevated plus maze (EPM) as described by Lapmanee et al. (2017). The apparatus was composed of two 48 cm × 12 cm open arms and two 48 cm × 48 cm × 12 cm closed arms. Open and closed arms were cross-shaped and the cross-center was a 5 cm × 5 cm open platform. The maze was 50 cm above the ground. Rats were placed on the central platform facing the open arm. During the 5-min period, the number of entries into every arm and the time spent in each arm was measured by observing the monitor above the maze.

#### Forced Swimming Test

We used the forced swimming test (FST) as described by Chen et al. (2020). This test was carried out twice: 10 min on the first day and 6 min on the second day. Rats were placed into a plexiglass barrel (diameter: 25 cm, height: 35 cm) filled with 25 cm water of room temperature (22 ± 1°C). The rats were dried immediately after the test and returned to home cage. The immobility time on the second day was calculated by a trained observer blind to grouping of the rats.

### Tissue Preparation

Within 2 h after FST, the rats were anesthetized with 10% chloral hydrate (0.4 ml/100 g body weight, intraperitoneal injection) and euthanized by decapitated. Brains were rapidly removed from the skulls. PFC was dissected, quickly frozen in liquid nitrogen and stored at −80°C until use. Half of PFC was used for biochemical analysis and the remaining half was used for western blotting analysis.

### Oxidative Stress Parameters

All PFC samples were homogenized individually by tissue protein extraction reagent (Pierce Biotechnology, Inc., Rockford, IL, United States) containing protease inhibitors. The homogenate was centrifuged at 12,000 × *g* for 10 min. Supernatant was used for the OS parameters, analyzed by a microplate reader (BioTek Instruments, Winooski, VT, United States). Commercial detection kits (Jiancheng Inc., Nanjing, China) were used to measure activity of superoxide dismutase (SOD) and MDA according to the manufacturer’ s instructions. Eight rats in each group were used for biochemistry analysis. Each determination was done in duplicate. All biochemical measures were normalized to the protein content.

### Western Blotting Analysis

Total protein was extracted from each PFC sample using RIPA lysis buffer, and the protein concentration was determined with a BCA protein assay kit. The protein extracts (30 μg per lane) were separated by SDS-PAGE and then transferred to polyvinylidene difluoride (PVDF) membranes, which were probed with v one of the following target primary antibodies at 4°C overnight: anti-ALDH-2 (1:1000, ab108306, Abcam, MA, United States); anti-4HNE (1:1000, ab46545, Abcam, MA, United States; OH, United States); anti-Caspase-3 (1:1000, AF6311, Affinity Biosciences, OH, United States); anti-cleaved caspase-3 (1:1000, AF7022, Affinity Biosciences); anti-BaX (1:1000, AF0120, Affinity Biosciences, OH, United States); anti-Bcl-2 (1:1000, AF6139, Affinity Biosciences, OH, United States); anti-HINT1 (1:1000, ab124912, Abcam, MA, United States); anti-PKCε (1:1000, Cell Signaling Technology Inc., MA, United States); anti-p-PKCε (1:1000, ab63387, Abcam, MA, United States); anti-β-actin (1:5000, C4, Santa Cruz Biotech, TX, United States). After incubating with the appropriate secondary antibodies for 1 h at room temperature, the membranes were treated with ECL reagents (Bio-Rad, Hercules, CA, United States), and the signals were visualized with an Odyssey Imaging System. The expression levels of specific protein were normalized to those of β-actin on the same PVDF membrane. The Quantity One software (Bio-Rad, Hercules, CA, United States) was used for quantification analysis. Five rats in each group were used for western blotting analysis.

### Statistical Analysis

All data were expressed in mean ± standard error of mean (SEM). SPSS13.0 software was used for data processing and analysis. Two-way analysis of variance (ANOVA) was performed to assess the main effects and the interaction of stress and administration of venlafaxine or NS, and one-way ANOVA was used for analyzing individual differences, followed by *post hoc* pairwise comparisons with Bonferroni corrections to the *P*-values (Li et al., 2019). The level of statistical significance was set at 0.05.

## Results

### Sucrose Preference Test

**FIGURE 2:**
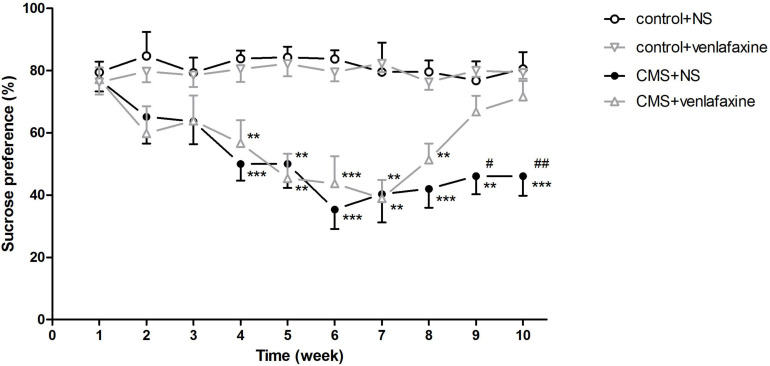
Sucrose preference test. Sucrose consumption in stressed rats decreased as the chronic mild stress procedure continued. Treatment with venlafaxine for 3 weeks increased sucrose preference of stressed rats. **P < 0.01, ***P < 0.001 compared with the control groups, ^#^*P* < 0.05, ^##^*P* < 0.01 compared with the CMS + venlafaxine group.

The one-way ANOVA showed that at baseline, no significant difference was found for sucrose preference among the four groups. Starting from 1 week of the CMS procedure, a continuous decrease in sucrose consumption was observed in stressed rats. Anhedonia was successfully induced after 3 weeks’ CMS regimen, showing predominantly lower amount of sucrose consumption than control group (*F*_3,__28_ = 10.670, *P* < 0.001). After administration of venlafaxine for 3 weeks, sucrose preference of the stressed rats was elevated to a similar level of the control rats (Figure 2).

The two-way ANOVA showed that at week 2 (*F*_1,28_ = 7.086, *P* = 0.013), 3 (*F*_1,28_ = 5.828, *P* = 0.023), 4 (*F*_1,28_ = 30.972, *P* < 0.001), 5 (*F*_1,28_ = 33.605, *P* < 0.001), 6 (*F*_1,28_ = 53.051, *P* < 0.001), 7 (*F*_1,28_ = 31.664, *P* < 0.001), 8 (*F*_1,28_ = 47.166, *P* < 0.001), 9 (*F*_1,28_ = 18.777, *P* < 0.001), and 10 (*F*_1,28_ = 18.046, *P* < 0.001), there was a significant main effect of stress on sucrose consumption. There was a significant main effect of venlafaxine on sucrose consumption at week 9 (*F*_1,28_ = 5.519, *P* = 0.026) and 10 (*F*_1,28_ = 6.020, *P* = 0.021). An interaction effect on sucrose consumption at week 10 (*F*_1,28_ = 7.267, *P* = 0.012) was indicated between stress and venlafaxine by two-way ANOVA analysis.

### Open Field Test

**FIGURE 3:**
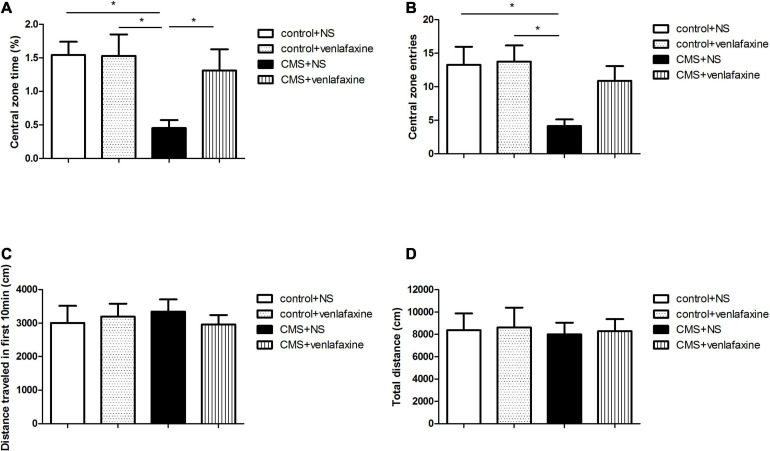
Open field test. **(A)** Percentage of time spent in the central area (*F*_3,36_ = 4.480, *P* = 0.011). **(B)** Central zone entries (*F*_3,36_ = 4.139, *P* = 0.015). **(C)** Total distance traveled in the first 10 min (*F*_3,36_ = 0.196, *P* = 0.899). **(D)** Total distance traveled in 1 h (*F*_3,36_ = 0.033, *P* = 0.992). *P < 0.05.

The one-way ANOVA showed that there was no significant difference among the 4 groups in the total distance traveled in the first 10 min (*F*_3,__28_ = 0.196, *P* = 0.899) or 1 h (*F*_3,__28_ = 0.033, *P* = 0.992). The decreased percentage of time spent in the central area (*F*_3,__28_ = 4.480, *P* = 0.011) and central zone entries (*F*_3,__28_ = 4.139, *P* = 0.015) demonstrated an anxiety-like behavior of rats in CMS+NS group (Figure 3).

The two-way ANOVA showed that there was a significant main effect of stress on percentage of time spent in the central area (*F*_1,28_ = 6.606, *P* = 0.016), and central zone entries (*F*_1,28_ = 7.588, *P* = 0.010). While there was no significant main effect of venlafaxine on OFT. No interaction effect between stress and venlafaxine was indicated by two-way ANOVA analysis.

### Elevated Plus Maze

**FIGURE 4:**
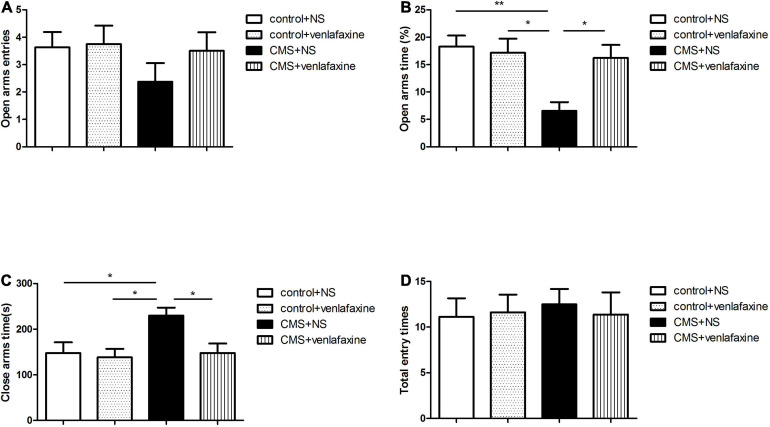
Elevated plus maze test. **(A)** Open arms entries. **(B)** Percentage of time spent in the open arms (*F*_3,36_ = 6.079, *P* = 0.003). **(C)** Time spent in closed arms (*F*_3,36_ = 4.496, *P* = 0.011). **(D)** Total number of entries (*F*_3,36_ = 0.086, *P* = 0.967). *P < 0.05, **P < 0.01.

The one-way ANOVA indicated that rats in the CMS + NS group showed an elevated level of anxiety as indicated by the decreased percentage of time spent in the open arms (*F*_3,__28_ = 6.079, *P* = 0.003), and the increase time spent in closed arms (*F*_3,__28_ = 4.496, *P* = 0.011), without change in the total number of entries (*F*_3,__28_ = 0.086, *P* = 0.967). These stress-induced anxiety-like behavioral alterations were reversed by continuous administration of venlafaxine (Figure 4).

The two-way ANOVA showed that there was a significant main effect of stress on percentage of time spent in the open arms (*F*_1,28_ = 8.375, *P* = 0.007), and time spent in closed arms (*F*_1,28_ = 5.150, *P* = 0.031). While there was no significant main effect of venlafaxine on EPM. An interaction effect on percentage of time spent in the open arms was indicated between stress and venlafaxine by two-way ANOVA analysis (*F*_1,28_ = 6.070, *P* = 0.020).

### Forced Swimming Test

**FIGURE 5:**
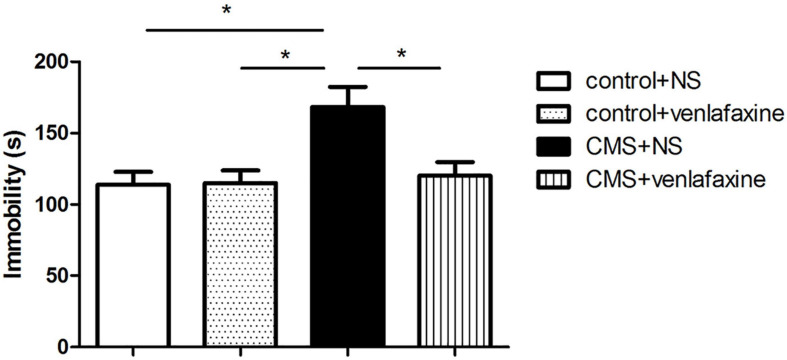
Forced swimming test. Rats in CMS + NS group showed longer immobility time (*F*_3,36_ = 6.035, *P* = 0.003). *P < 0.05.

The one-way ANOVA showed that immobility time was predominantly longer in stressed rats (*F*_3,__28_ = 6.035, *P* = 0.003), which was related to depressive behavior. The depression-like behavior was ameliorated by continued treatment with venlafaxine (Figure 5).

The two-way ANOVA showed that there was a significant main effect of stress on immobility time (*F*_1,28_ = 7.854, *P* = 0.009). There was a significant main effect of venlafaxine on immobility time (*F*_1,28_ = 4.883, *P* = 0.035). An interaction effect between stress and venlafaxine was indicated by two-way ANOVA analysis (*F*_1,28_ = 5.369, *P* = 0.028).

### The Level of Biomarkers of Oxidative Stress in the PFC

**FIGURE 6:**
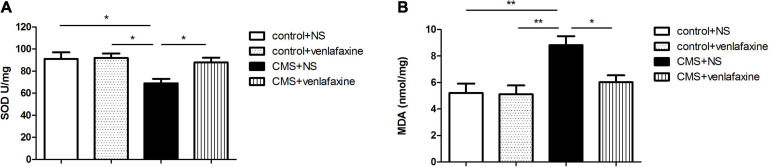
Levels of oxidative stress-related biomarkers in prefrontal cortex. **(A)** SOD (*F*_3,36_ = 5.305, *P* = 0.005), **(B)** MDA (*F*_3,36_ = 7.271, *P* = 0.001) content in PFC. *P < 0.05, **P < 0.01.

The antioxidant capacity in the PFC was determined by measuring the levels of activities of SOD, and the status of lipid oxidation was determined by measuring levels of MDA. The one-way ANOVA showed that CMS elicited a significant decrease of SOD (*F*_3,__28_ = 5.305, *P* = 0.005), and a marked increase MDA (*F*_3,__28_ = 7.271, *P* = 0.001) levels in rat PFC, compared with those in the control + NS and control + venlafaxine groups. Meanwhile, venlafaxine treatment effectively increased SOD levels and decreased MDA levels caused by CMS (Figure 6).

The two-way ANOVA showed that there was a significant main effect of stress on the levels of SOD (*F*_1,28_ = 7.767, *P* = 0.009) and MDA (*F*_1,28_ = 12.384, *P* = 0.001). There was a significant main effect of venlafaxine on the levels of SOD (*F*_1,28_ = 4.457, *P* = 0.044) and MDA (*F*_1,28_ = 5.035, *P* = 0.033). An interaction effect on the levels of MDA was indicated between stress and venlafaxine by two-way ANOVA analysis (*F*_1,28_ = 4.395, *P* = 0.045).

### Relative Protein Levels of ALDH-2/4HNE/Caspase-3/Cleaved Caspase-3/Bax/Bcl-2 in the PFC

**FIGURE 7:**
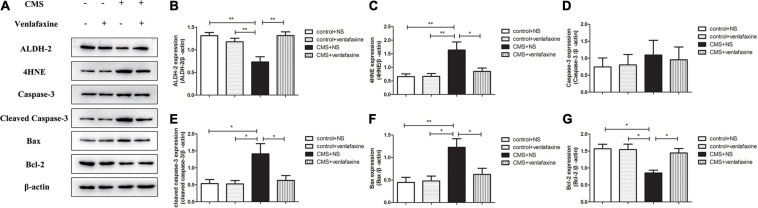
Relative protein levels of ALDH-2/4HNE/Caspase-3/cleaved caspase-3/Bax/Bcl-2 in the prefrontal cortex of CMS rats. **(A)** Representative western blot bands. **(B–G)** The relative protein expressions of ALDH-2 (*F*_3,16_ = 9.839, *P* = 0.001), 4HNE (*F*_3,16_ = 6.776, *P* = 0.004), Caspase-3 (*F*_3,16_ = 0.199, *P* = 0.895), cleaved caspase-3 (*F*_3,16_ = 5.436, *P* = 0.009), Bax (*F*_3,16_ = 6.609, *P* = 0.004), and Bcl-2 (*F*_3,16_ = 6.705, *P* = 0.004) are expressed as the optical density ratio of a target protein to β-actin. *P < 0.05, **P < 0.01.

The one-way ANOVA showed that protein levels of ALDH-2 (*F*_3,16_ = 9.839, *P* = 0.001) and Bcl-2 (*F*_3,16_ = 6.705, *P* = 0.004) were significantly decreased in PFC of CMS + NS group compared with the control groups. Levels of 4HNE (*F*_3,16_ = 6.776, *P* = 0.004) and Bax (*F*_3,16_ = 6.609, *P* = 0.004) were significantly increased in the CMS + NS group. There was no significant difference in levels of caspase-3 between groups (*F*_3,16_ = 0.199, *P* = 0.895), while the levels of cleaved caspase-3 were significantly increased in the CMS group (*F*_3,16_ = 5.436, *P* = 0.009). The administration of venlafaxine increased the expression of ALDH-2 and Bcl-2, and decreased the expression of 4HNE, cleaved caspase-3 and Bax in PFC of CMS rats, which was similar with the control group (Figure 7).

The two-way ANOVA showed that there was a significant main effect of stress on the levels of ALDH-2 (*F*_1,16_ = 6.331, *P* = 0.023), 4HNE (*F*_1,16_ = 10.610, *P* = 0.005), cleaved caspase-3 (*F*_1,16_ = 7.148, *P* = 0.017), Bax (*F*_1,16_ = 10.712, *P* = 0.005), and Bcl-2 (*F*_1,16_ = 9.800, *P* = 0.006). There was a significant main effect of venlafaxine on the levels of ALDH-2 (*F*_1,16_ = 6.494, *P* = 0.021), 4HNE (*F*_1,16_ = 4.766, *P* = 0.044), cleaved caspase-3 (*F*_1,16_ = 4.709, *P* = 0.045), and Bcl-2 (*F*_1,16_ = 4.473, *P* = 0.045). An interaction effect on the levels of ALDH-2 (*F*_1,16_ = 16.692, *P* = 0.001), 4HNE (*F*_1,16_ = 4.952, *P* = 0.041), Bax (*F*_1,16_ = 5.059, *P* = 0.039), and Bcl-2 (*F*_1,16_ = 5.573, *P* = 0.031) was indicated between stress and venlafaxine by two-way ANOVA analysis.

### Relative Protein Levels of HINT1/PKC ε/p-PKC ε in the PFC

**FIGURE 8:**
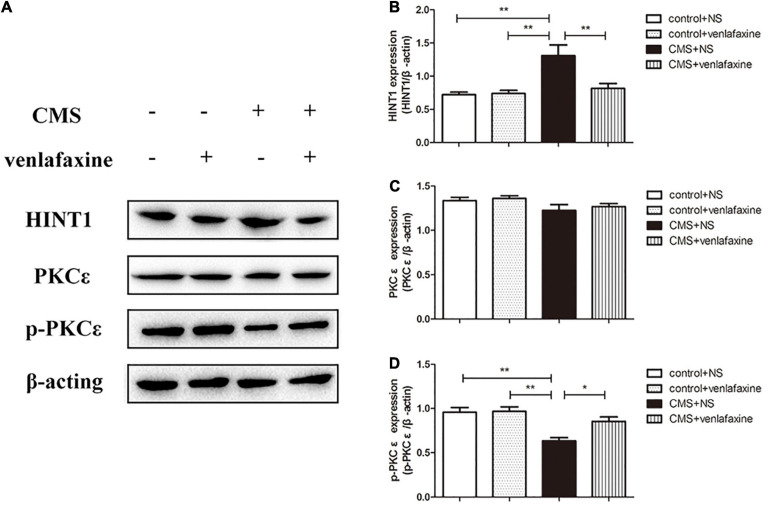
Relative protein levels of HINT1/PKCε/p-PKCε in the prefrontal cortex of CMS rats. **(A)** Representative western blot bands. **(B–D)** The relative protein expressions of HINT1 (*F*_3,16_ = 8.766, *P* = 0.001), PKC ε (*F*_3,16_ = 1.881, *P* = 0.174) and p-PKC ε (*F*_3,16_ = 10.087, *P* = 0.001) are expressed as the optical density ratio of a target protein to β-actin. *P < 0.05, **P < 0.01.

The one-way ANOVA showed that protein levels of HINT1 were significantly increased in PFC of CMS + NS group compared with the control groups (*F*_3,16_ = 8.766, *P* = 0.001). There was no significant difference in levels of PKC ε between groups (*F*_3,16_ = 1.881, *P* = 0.174); however, the phosphorylated forms of PKC ε were significantly decreased in the CMS + NS group (*F*_3,16_ = 10.087, *P* = 0.001). The administration of venlafaxine decreased the expression of HINT1 and increased the expression of p-PKC ε in PFC of CMS rats, which was similar with the control group (Figure 8).

The two-way ANOVA showed that there was a significant main effect of stress on the levels of HINT1 (*F*_1,16_ = 12.458, *P* = 0.003), PKC ε (*F*_1,16_ = 5.041, *P* = 0.039) and phosphorylated forms of PKC ε (*F*_1,16_ = 20.056, *P* < 0.001). There was a significant main effect of venlafaxine on the levels of HINT1 (*F*_1,16_ = 6.437, *P* = 0.022) and phosphorylated forms of PKC ε (*F*_1,16_ = 5.602, *P* = 0.031). An interaction effect on the levels of HINT1 (*F*_1,16_ = 7.404, *P* = 0.015) and phosphorylated forms of PKC ε (*F*_1,16_ = 4.604, *P* = 0.048) was indicated between stress and venlafaxine by two-way ANOVA analysis.

## Discussion

In the current study, we have investigated the CMS model in terms of face validity (phenomenological similarities between the model and the disorder), construct validity (a sound theoretical rationale), and predictive validity (basically correspondence of drug effects in the model and in the clinic) (Willner, 1997).

For face validity, the results of OFT, EPM, and FST revealed that stressed rats displayed elevated level of anxiety and depression compared with the controls. Decreased sucrose preference among stressed rats is indicative of a loss of interest in normally pleasurable and rewarding activities. Such anhedonic behavior is considered as a core symptom and corroborative evidence of depression (Ma et al., 2011, 2016). Those changes in behavioral tests suggested that the model of CMS was successfully established, which was accordant with other studies (Willner, 1997; Xing et al., 2013; Ge et al., 2015).

For construct validity, we found the CMS rats had elevated expression of HINT1, decreased levels of p-PKC ε and ALDH-2, and accumulated 4HNE in PFC. Moreover, CMS increased the levels of cleaved caspase-3 and Bax, and decreased the level of Bcl-2 in PFC. These results indicated that HINT1 was involved in the CMS elicited OS and apoptosis in PFC, probably through the PKC ε/ALDH-2/4HNE pathway.

In biochemical tests, we measured SOD level, which is usually used to reflect OS. When the antioxidant such as SOD is decreased, a large number of ROS will result in lipid peroxidation. As a product of lipid peroxidation, MDA is used to reflect OS injuries. In agreement with previous findings in brain (Lindqvist et al., 2017), we observed that CMS elicited significant changes in indicators of OS, including the decrease of SOD and the increase of MDA. Moreover, studies (Ozcan et al., 2004; Lindqvist et al., 2017) and meta-analysis (Jimenez-Fernandez et al., 2015) investigating the OS profile found a reduction in antioxidant enzymes and elevation of the products of lipid peroxidation in the blood of patients with MDD.

Histidine triad nucleotide binding protein 1 was first supposed to be PKC inhibitor 1, and then found to be a haplo-insufficient tumor suppressor protein (Li et al., 2006; Dang et al., 2017). In frontal cortices of Hint1−/− mice, the PKC enzymatic activity was significantly increased (Garzón-Niño et al., 2017). Besides, both Hint1−/− and Hint1+/− mice exhibit an elevated rate of spontaneous tumor development, and enhanced susceptibility to tumor induction by 7,12-dimethylbenzanthracene (Li et al., 2006). Generally, the altered expression of tumor suppressor proteins is associated with abnormal induction of apoptosis. After transient transfection with Hint1 by plasmids, human SW480 colon cancer cells and human MCF-7 breast carcinoma cells underwent apoptosis as analyzed by the expression of pro-caspase-3 and poly (ADP-ribose) polymerase cleavage (Weiske and Huber, 2006). In Hint1-transfected SW480 and MCF-7 cells, the expression of p53 at both the mRNA and protein level was up-regulated, which was demonstrated to be an upstream inducer of Bax expression (Yu and Zhang, 2005). Meanwhile, in response to the knockdown of Hint1 by short hairpin RNA, down-regulation of p53 and Bax expression was also observed (Yu and Zhang, 2005). In addition, HINT1 could be involved in the mood and behavior regulations, which was found in previous studies. Sun et al. (2017) found that both male and female HINT1 knockout and heterozygosity mice had a trend of anxiolytic-like behavior and antidepression-like behavior compared with control group. Both male and female HINT1 knockout mice showed elevated antidepression-like behavior under chronic immobilization stress. Zhou et al. (2018) found that exposure to constant light and/or darkness can induce significant changes in affective and cognitive responses in C57BL/6 mice, possibly through HINT1-induced activation of apoptotic pathways. However, there are few studies reported the relationship between HINT1 and OS/ROS in depression.

The role of PKC signaling system in the pathophysiology and treatment of mood disorders was found in previous studies (Abrial et al., 2011). According to the structure and requirements for activation, PKC family is divided into three categories, conventional PKCs (PKC α, PKC β I, PKC β II, and PKC γ), novel PKCs (PKC δ, PKC ε, PKC η, and PKC θ) and atypical PKCs (PKC λ and ζ) (Ohno and Nishizuka, 2002). Through their phosphorylation, PKCs are able to modulate various neuronal functions (Obis et al., 2015). PKC ε is highly expressed in brain regions participant in mood regulation such as hippocampus and PFC, and plays a role of neurotransmitter release, cell proliferation, and synaptic remodeling (Abrial et al., 2011). Intriguingly, phosphorylation of PKC ε was proposed as an upstream regulatory molecule of ALDH2 alleviating OS and neuronal apoptosis (Zuo et al., 2019). 4-HNE is a stable product of lipid peroxidation that acts as a key mediator of OS-induced cytotoxic effects, while ALDH2 serves as an endogenous shield against OS-mediated damage by clearing 4-HNE (Sidramagowda Patil et al., 2020). Correspondingly, in this study, we found in PFC of CMS rats, high expression of HINT1 was accompanied with reduced expression of p- PKC ε and ALDH-2, while 4HNE was over-accumulated. In addition, we also found CMS increased the levels of cleaved caspase-3 and Bax, and decreased the level of Bcl-2 in PFC. Cleaved caspase-3 is a widely accepted apoptosis executor. Bcl-2 suppress while Bax promotes neuronal apoptosis, which is involved in the onset of depression (Kubera et al., 2011).

For predictive validity, we found the depression-like and anhedonia-like behavior was significantly ameliorated by chronic administration of venlafaxine. The possible mechanism may include that venlafaxine can prevent the down-regulation of neurogenesis and cell proliferation (Xu et al., 2006). The ability to reduce OS, inflammation and apoptosis of venlafaxine was demonstrated in lung tissue (El-Kashef, 2018), kidney (El-Kashef and Sharawy, 2018), and brain microvascular endothelial cells (Lv et al., 2014). In the present study, the level of biomarkers of OS, expression of apoptosis-related protein, as well as the expression of HINT1 in PFC of CMS rats was reversed after the chronic administration of venlafaxine. However, the effect of antidepressants on apoptosis appears to be more complex. The contrary studies showing pro-apoptotic activity of antidepressants in tumorigenic cell lines suggest that the pro-apoptotic or anti-apoptotic effect might be contingent on cell type and culture conditions (Xia et al., 1999; Drzyzga et al., 2009).

## Conclusion

In conclusion, we found anhedonia-like and depressive-analog changes in behavior of rats, as well as the elevated OS and apoptosis in PFC after CMS. The involvement of HINT1 protein through PKC ε/ALDH-2/4HNE pathway in CMS was firstly demonstrated. Moreover, these alterations in behavior and molecule were prevented by antidepressant venlafaxine. The results suggest that the suppression of HINT1 might have potential as a novel therapeutic strategy for depression. Nevertheless, several certain limitations should be considered when viewing these data. Firstly, to avoid the impact of fluctuating sex hormone, only male rats were adopted in the present. However, female has higher incidence of mood disorders than males in humans. Secondly, it is controversial that the comparability between CMS in rodents and psychosocial stress in humans. And the results in animals are difficult to transfer in human. Thirdly, only venlafaxine was administrated, and whether these results could be generalized to other antidepressant agents such as SSRIs needs to be clarified in the future. In addition, some confounding factors should not be ignored. For example, different housing conditions for control and stressed animals might have impact on the outcome of this study. Only 2 hours’ interval between the end of FST and tissue collection might affect biochemical changes of rats, even though behavioral tests might be a secondary factor. Hence, the results should be interpreted with caution.

## Data Availability Statement

The original contributions presented in the study are included in the article/supplementary material, further inquiries can be directed to the corresponding author/s.

## Ethics Statement

The animal study was reviewed and approved by the Xi’an Jiaotong University Laboratory Animal Administration Committee.

## Author Contributions

FL and Y-HD conceived and designed the experiments. FL, Y-YD, GL, and PL performed the experiments. YZ performed the statistical analyses and interpretation of the data. FL drafted the manuscript. Y-HD revised the manuscript. All authors have read and approved the final manuscript.

## Conflict of Interest

The authors declare that the research was conducted in the absence of any commercial or financial relationships that could be construed as a potential conflict of interest.
